# Discordance Between Eye-Specific Clinical Outcomes and Patient-Reported Visual Function After Faricimab Treatment for Neovascular Age-Related Macular Degeneration: A Two-Case Report

**DOI:** 10.3390/vision10040060

**Published:** 2026-08-26

**Authors:** Tereza Pařilová, Libor Hejsek, Lenka Hodačová

**Affiliations:** 1 Faculty of Medicine in Hradec Králové, Department of Preventive Medicine, Charles University, Šimkova 870, 500 03 Hradec Kralove, Czech Republic; hodacl@lfhk.cuni.cz; 2 Eye Clinic, University Hospital Hradec Králové, Sokolská 581, 500 05 Hradec Kralove, Czech Republic; libor.hejsek@fnhk.cz; 3 Faculty of Medicine in Hradec Králové, Eye Clinic, Charles University, 500 03 Hradec Kralove, Czech Republic

**Keywords:** age-related macular degeneration, quality of life, patient-reported outcome measures, visual acuity, colour perception, treatment outcome, case report

## Abstract

Neovascular age-related macular degeneration is a retinal disease of older adults causing central vision loss associated with macular neovascularization. Treatment response is commonly evaluated using visual acuity and retinal morphology. These measures may not fully reflect patient-perceived benefit. We report two older adults with neovascular age-related macular degeneration treated with faricimab who demonstrated discordance between clinical and patient-reported outcomes. In the first case, best-corrected visual acuity changed from 20/200 to 20/180 and central retinal thickness decreased from 480 to 389 µm, whereas the National Eye Institute Visual Function Questionnaire–25 score increased from 33.87 to 60.99 and the exploratory ordinal colour-vividness rating was 25 at baseline and 100 at follow-up. In the second case, best-corrected visual acuity improved from 20/100 to 20/45 and central retinal thickness decreased from 341 to 186 µm, whereas the National Eye Institute Visual Function Questionnaire–25 score declined from 67.08 to 33.17 and the exploratory ordinal colour-vividness rating was 75 at baseline and 25 at follow-up. Changes in patient-reported visual functioning may not always parallel conventional clinical outcomes, while the contrasting exploratory colour-vividness ratings observed in these two cases warrant further investigation.

## 1. Introduction

Neovascular age-related macular degeneration (nAMD) predominantly affects older adults and may substantially interfere with independence, daily functioning, social participation, and quality of life (QoL). The disease represents a growing public health challenge worldwide and affects aging populations across diverse geographic and ethnic backgrounds [1,2,3]. Although anti-vascular endothelial growth factor (anti-VEGF) therapy has improved the management of this condition, treatment response is still commonly evaluated using visual acuity and retinal morphology assessed by optical coherence tomography (OCT) [4,5,6].

These objective measures are essential for monitoring disease activity, but they may not fully reflect the aspects of vision that determine how older patients experience everyday life [1,7]. In this context, patient-reported outcome measurements (PROMs) may provide complementary information about perceived treatment benefit, functional adaptation, and vision-related well-being [1,3,7,8]. For many older adults, maintaining independence, confidence in everyday activities, and social engagement may be as important as improvements in disease-specific clinical measures [8,9,10]. Despite substantial advances in anti-VEGF therapy, whether conventional clinical outcomes adequately reflect treatment success from the patient’s perspective remains an open question [3,11,12]. These observations have largely been derived from standardized vision-related QoL instruments that quantify predefined domains of visual functioning and daily activities [1,11,12]. Importantly, these instruments assess patients’ overall visual functioning and vision-related quality of life in everyday life rather than eye-specific visual performance [10,13].

Nevertheless, some aspects of subjective visual experience remain difficult to quantify and are not routinely assessed in clinical trials or everyday practice. Colour vision represents a broader aspect of visual function, whereas subjective colour vividness refers specifically to the perceived intensity or vividness of colours [14]. Although the National Eye Institute Visual Function Questionnaire–25 (NEI VFQ-25) includes a single item addressing colour vision, it does not specifically evaluate the perceived intensity or vividness of colours and may therefore provide only limited insight into this aspect of visual experience [14,15].

We present two contrasting cases illustrating how clinical outcomes, vision-related quality of life, and exploratory colour vividness may follow markedly different trajectories during anti-VEGF treatment for nAMD. The cases highlight discordance between conventional clinical outcomes and patient-reported visual functioning, alongside contrasting patterns in the exploratory colour-vividness ratings.

## 2. Participants and Methods

### 2.1. Participants

This report describes two older adults in their seventies with treatment-naïve nAMD who underwent intravitreal faricimab therapy at the Eye Clinic of the University Hospital Hradec Králové, Czech Republic. In both cases, treatment-naïve nAMD was diagnosed based on clinical examination, OCT imaging, and the patients’ self-reported visual symptoms. The diagnosis was supported by the combined findings of OCT, Amsler grid assessment, and dilated fundus examination. Alternative causes of macular fluid were considered clinically; central serous chorioretinopathy and polypoidal choroidal vasculopathy were considered unlikely based on the patients’ age and clinical presentation, with no history of corticosteroid use, while diabetic macular edema was excluded by the absence of diabetes.

The present report describes two treatment-naïve patients retrospectively selected from a prospective cohort of 19 treatment-naïve patients receiving intravitreal faricimab. Patient-reported outcomes, including the NEI VFQ-25 and exploratory ordinal colour-vividness rating, were prospectively collected from all patients in the cohort. Following completion of follow-up, the two cases were intentionally selected because they demonstrated contrasting discordance between conventional clinical outcomes and patient-reported outcomes. They are presented as illustrative, hypothesis-generating examples.

Clinical outcomes, including best-corrected visual acuity (BCVA), optical coherence tomography (OCT), and central retinal thickness (CRT), were assessed in the treated eye. BCVA was measured using a Snellen visual acuity chart following objective autorefraction and subsequent subjective refinement using spherical and cylindrical trial lenses. Testing was performed at the standard distance of 6 m whenever possible. In patients with poorer visual acuity, the testing distance was reduced as required, and the measured Snellen fraction was subsequently converted to its equivalent fraction at the standard testing distance; thus, the value of 20/180 reported in Case 1 represents a standardized Snellen equivalent derived from testing at a reduced distance. The same established clinical testing protocol and examiner were used throughout follow-up.

Patient-reported outcomes were assessed using the NEI VFQ-25, which was administered according to the standard questionnaire instructions and therefore reflected patients’ overall visual functioning in daily life rather than the treated eye alone. Both affected eyes were phakic. Assessments were performed at baseline and after completion of six intravitreal faricimab injections. Both patients received four initial monthly faricimab injections followed by an individualized treat-and-extend regimen. After the loading phase, treatment intervals were extended or shortened according to disease activity based on OCT findings, including retinal fluid and CRT, slit lamp examination, and the treating ophthalmologist’s overall clinical assessment. The different timing of the sixth injection in Case 1 (Week 26) and Case 2 (Week 32) reflected individualized interval adjustment according to the clinical and OCT course. Both patients completed all six scheduled intravitreal faricimab injections without treatment discontinuation, and treatment was well tolerated, with no ocular or systemic adverse events or serious adverse events reported during follow-up.

### 2.2. Clinical Outcome Assessment

BCVA was assessed using the Snellen visual-acuity protocol described above, with reduced distance testing and conversion to the corresponding standard distance Snellen equivalent when required.

OCT imaging was performed using the Spectralis OCT system (Heidelberg Engineering GmbH, Heidelberg, Germany), with the same device used throughout follow-up. Retinal thickness values were obtained directly from the device-generated retinal thickness analysis. Automated segmentation was reviewed by the same ophthalmologist, and no manual segmentation correction was performed. OCT images were included when considered clinically interpretable by the examining ophthalmologist. Retinal morphology was evaluated from the OCT images as part of the clinical assessment.

### 2.3. Patient-Reported Outcome Assessment

Vision-related QoL was assessed using the NEI VFQ-25, one of the most widely used and validated PROMs in ophthalmology. A Czech translation derived from the validated Slovak version of the NEI VFQ-25 was used. This Czech version had previously been used in a published study by Nekolová et al. [16]. No modifications to the questionnaire content, response options, or recall periods were made. The questionnaire evaluates the impact of visual impairment on patients’ daily functioning across multiple domains, including general vision, near activities, distance activities, social functioning, mental health, role difficulties, dependency, driving, colour vision, peripheral vision, and ocular pain. Responses are transformed to a 0–100 scale, with higher scores indicating better patient-perceived visual functioning and vision-related QoL. The composite score was calculated as the mean of the vision-targeted subscales according to the original scoring algorithm [14], excluding the General Health subscale, which is not included in the composite score. Because neither patient had been driving before study enrolment for reasons unrelated to nAMD, the Driving subscale was treated as not evaluated and was excluded from the composite score calculation in accordance with the NEI VFQ-25 scoring recommendations. The NEI VFQ-25 was interviewer-administered by the same trained study staff member under the same conditions at both assessments because the patients’ age and visual impairment could make independent reading of the questionnaire difficult. Questionnaire administration followed the clinical examination; however, patients were not informed of their numerical BCVA or OCT results before completing the questionnaire, and the interviewer recording the responses was masked to these clinical results.

To complement the NEI VFQ-25, patients additionally rated their colour vividness using a single exploratory, non-validated patient-reported question. The question was designed to explore patients’ subjective perception of colour vividness as part of their overall visual experience, an aspect not specifically addressed by the NEI VFQ-25. The item did not undergo formal psychometric validation and should be interpreted as an exploratory ordinal measure. The same question was prospectively administered to all patients in the study cohort at each assessment, with the reference point adapted to the assessment time. At baseline, immediately before treatment initiation, patients were asked: “Compared with how you remember seeing before your vision worsened due to the current macular disease, how do you currently perceive the colours of objects and your surroundings in terms of their vividness, clarity, saturation, contrast, and ability to distinguish between similar colours?” At follow-up, patients were asked: “Compared with before treatment, how do you currently perceive the colours of objects and your surroundings in terms of their vividness, clarity, saturation, contrast, and ability to distinguish between similar colours?”. Thus, the baseline rating reflected patients’ perceived change associated with the preceding visual deterioration, whereas the follow-up rating reflected their perceived change relative to their pre-treatment visual experience.

The question was interviewer-administered by the same examiner at both study visits. At baseline, responses were recorded using a five-category ordinal response format (0 = much worse, 25 = markedly worse, 50 = moderately worse, 75 = slightly worse, and 100 = unchanged), reflecting patients’ current subjective colour experience relative to their recalled vision before the preceding visual deterioration. At follow-up, responses were recorded using a five-category ordinal response format (0 = much worse, 25 = worse, 50 = unchanged, 75 = noticeably better, and 100 = markedly better), reflecting patients’ current subjective colour experience relative to their pre-treatment state. The response format was chosen to maintain consistency with the preceding NEI VFQ-25 assessment and to minimise additional cognitive burden in this elderly population with visual impairment. The numerical values represented ordered response categories and were not assumed to constitute an equal-interval or continuous metric. The examiner was masked to the clinical outcomes at the time of questionnaire administration. Accordingly, baseline and follow-up ratings were interpreted descriptively according to their respective reference points rather than as measurements on a continuous change scale.

### 2.4. Ethics

The study was approved by the Ethics Committee of the University Hospital Hradec Králové (approval No. 202308 P02, date 17 August 2023). Written informed consent for participation and publication of anonymized clinical data and retinal images was obtained from both patients. Permission to conduct the study and publish the findings was granted by the Head of the Participating Department. The study was conducted in accordance with the ethical principles of respect for persons, beneficence, and justice as outlined in the Belmont Report and complied with the Declaration of Helsinki and its subsequent amendments.

## 3. Case Presentation

### 3.1. Case 1

A woman in her seventies presented with progressive visual decline in the right eye over several months. Initially, she attributed her symptoms to refractive error, but they did not improve with spectacle correction. She reported progressive central visual distortion. Her medical history was notable only for treated and stable arterial hypertension.

At presentation, BCVA was 20/200 in the right eye. The fellow eye remained clin-ically stable throughout follow-up, with a BCVA of 20/25 and no evidence of retinal, lenticular, ocular surface, neurological, or other clinically relevant ocular pathology that could reasonably account for the observed changes in patient-reported outcomes. During follow-up, no clinically relevant progression of lens opacity, ocular-surface disease, glaucoma, or other ocular condition potentially affecting patient-reported visual functioning was observed. No new neurological or systemic illness, depressive or cognitive symptoms, changes in medication, mobility or social circumstances, or treatment-related burden, anxiety, pain, or other new symptoms were reported by the patient.

**Figure 1:**
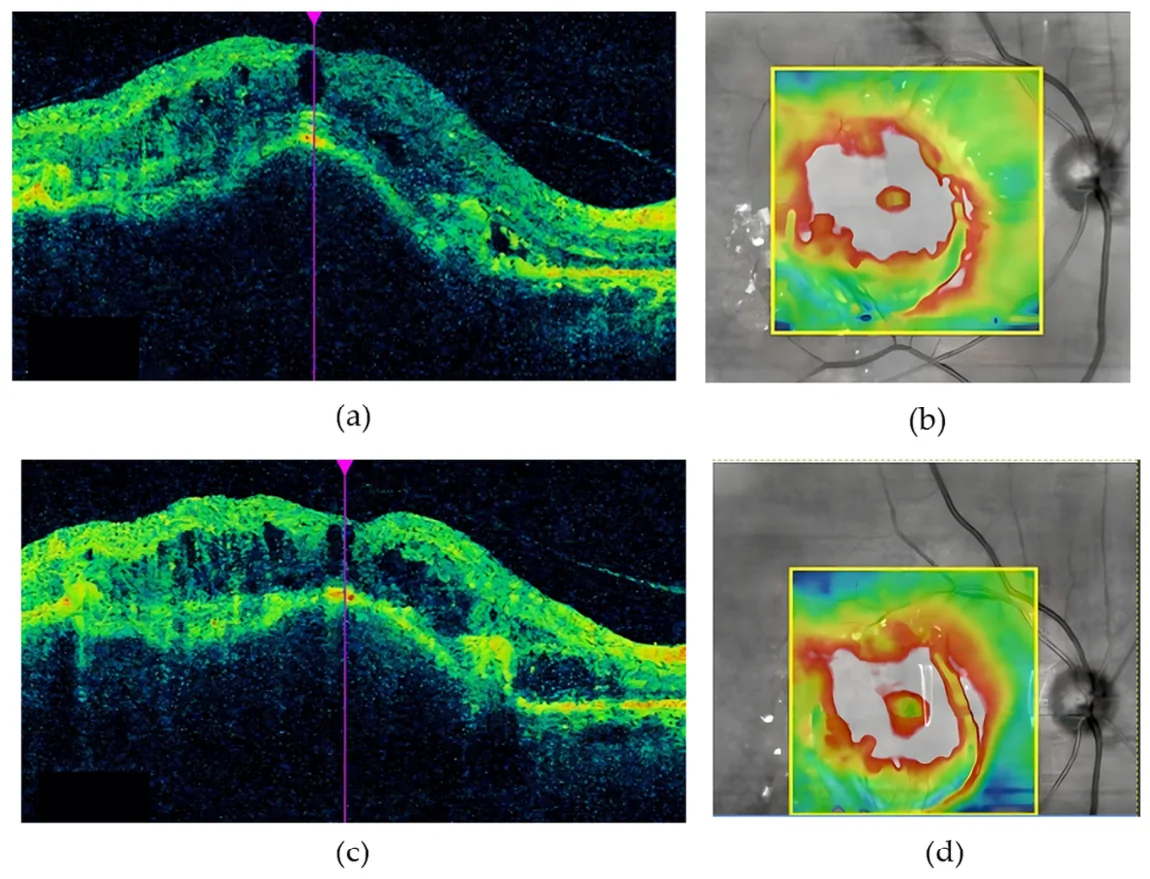
**Retinal imaging of Case 1 at baseline and after six intravitreal faricimab injections.** (**a**) Baseline OCT demonstrating marked structural abnormalities of the macula with retinal thickening (CRT, 480 µm); (**b**) corresponding baseline retinal map; (**c**) OCT after six intravitreal faricimab injections, demonstrating persistent structural abnormalities with a reduction in CRT to 389 µm; (**d**) corresponding retinal map after six injections. Patient-identifying information was cropped from the original images. Original colour images exported directly from the OCT device at the highest available quality are presented without image processing that could alter or reconstruct retinal structures. CRT, central retinal thickness; OCT, optical coherence tomography.

Fundus examination revealed extensive submacular haemorrhage involving the foveal region. OCT demonstrated marked structural abnormalities of the macula with retinal thickening and fluid-associated changes, with a CRT of 480 µm (Figure 1a,b). At the treatment initiation visit, after partial spontaneous resorption of the submacular haemorrhage, baseline BCVA and OCT were assessed before the first intravitreal faricimab injection. The initial patient-reported assessment was performed within several days of treatment initiation. Following six intravitreal faricimab injections administered over 26 weeks, BCVA changed from 20/200 to 20/180. OCT demonstrated persistent structural abnormalities, with CRT decreasing from 480 µm to 389 µm (Figure 1c,d).

At final follow-up, the fellow eye remained clinically stable, with a BCVA of 20/25^−2^ and no evidence of retinal, lenticular, or other clinically relevant ocular pathology.

Despite persistently poor BCVA and residual structural abnormalities on OCT, patient-reported visual functioning improved substantially. The NEI VFQ–25 composite score increased from 33.87 at baseline to 60.99 after the sixth injection. The greatest improvements were observed in role difficulties, mental health, and general vision, while anatomical improvement remained limited. At baseline, the exploratory ordinal colour-vividness rating was 25, indicating markedly worse subjective colour experience relative to the patient’s recalled vision before the preceding visual deterioration. After the sixth injection, the rating was 100, indicating markedly better subjective colour experience relative to the pre-treatment state. The NEI VFQ-25 item contributing to the Color Vision subscale (item 12) changed from 50 at baseline to 75 after the sixth injection. When asked to describe the overall change in her visual experience, the patient replied, “Like night and day.” This statement accompanied the observed improvement in patient-reported visual functioning and the exploratory colour vividness rating, while improvement in treated eye BCVA and retinal morphology remained limited.

### 3.2. Case 2

A man in his seventies presented with gradually worsening vision in the right eye that did not improve with spectacle correction. Symptoms developed over several weeks to a few months. He reported progressive central visual blurring and distortion affecting reading and near activities. His medical history included treated arterial hypertension. The fellow eye showed no signs of nAMD. No clinically relevant progression of lenticular opacity, ocular-surface disease, glaucoma, or other ocular condition that could account for the deterioration in patient-reported visual functioning was identified during follow-up. The fellow eye remained clinically stable. The patient did not report new neurological or systemic illness, depressive or cognitive symptoms, changes in medication, mobility or social circumstances, or treatment-related burden, anxiety, pain, or other new symptoms during follow-up.

**Figure 2:**
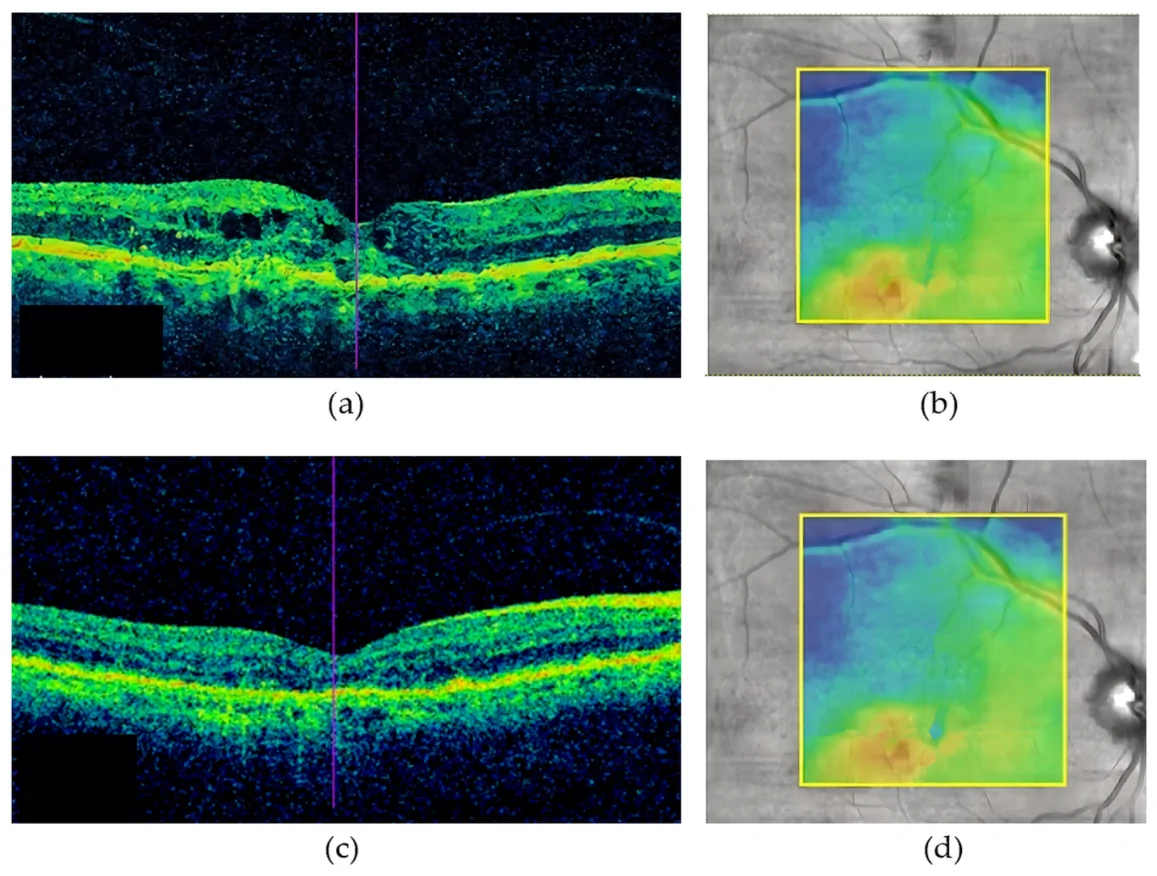
**Retinal imaging of Case 2 at baseline and after six intravitreal faricimab injections.** (**a**) Baseline OCT demonstrating marked structural abnormalities of the macula with retinal thickening (CRT, 341 µm); (**b**) Corresponding baseline retinal map; (**c**) OCT after six intravitreal faricimab injections, demonstrating a marked reduction in retinal thickening and structural abnormalities, with CRT decreasing to 186 µm, although residual structural abnormalities remained; (**d**) Corresponding retinal map after six injections. Patient-identifying information was cropped from the original images. Original colour images exported directly from the OCT device at the highest available quality are presented without image processing that could alter or reconstruct retinal structures. CRT, central retinal thickness; OCT, optical coherence tomography.

At presentation, BCVA was 20/100 in the right eye. The fellow eye had a BCVA of 20/25 and demonstrated age-appropriate anterior and posterior segment findings, with no evidence of nAMD or other clinically relevant ocular pathology. Fundus examination of the affected eye suggested exudative macular pathology. OCT demonstrated marked structural abnormalities of the macula with retinal thickening, with a CRT of 341 µm (Figure 2a,b). Intravitreal faricimab therapy was initiated after diagnosis. Following six intravitreal faricimab injections administered over 32 weeks, BCVA improved from 20/100 to 20/45. OCT demonstrated a marked reduction in retinal thickening and structural abnormalities, with CRT decreasing from 341 µm to 186 µm, although residual structural abnormalities remained (Figure 2c,d).

At final follow-up, the fellow eye remained clinically stable, with a BCVA of 20/25^−1^ and no evidence of retinal, lenticular, or other clinically relevant ocular pathology.

Despite favourable functional and anatomical outcomes, patient-reported visual functioning declined substantially, with the greatest declines observed in social functioning, role difficulties, dependency, and colour vision.

At baseline, the exploratory ordinal colour-vividness rating was 75, indicating slightly worse subjective colour experience relative to the patient’s recalled vision before the preceding visual deterioration. After the sixth injection, the rating was 25, indicating worse subjective colour experience relative to the pre-treatment state. The NEI VFQ-25 item contributing to the Color Vision subscale (item 12) changed from 100 at baseline to 50 after the sixth injection.

When asked to describe the overall change in his visual experience, the patient replied, “Things had faded.” This statement accompanied the observed deterioration in patient-reported visual functioning and the exploratory colour-vividness rating, while visual acuity and retinal morphology improved substantially.

The clinical and patient-reported outcomes of both cases at baseline and final follow-up are summarized in Table 1. Complete baseline and follow-up NEI VFQ-25 subscale scores for both cases are provided in Supplementary Table S1.

In both cases, the patterns observed in the exploratory colour-vividness ratings were consistent with the direction of change in the NEI VFQ-25 composite score. However, the exploratory colour-vividness ratings did not fully correspond to the changes observed in the single colour vision item of the NEI VFQ-25.

**Figure 3:**
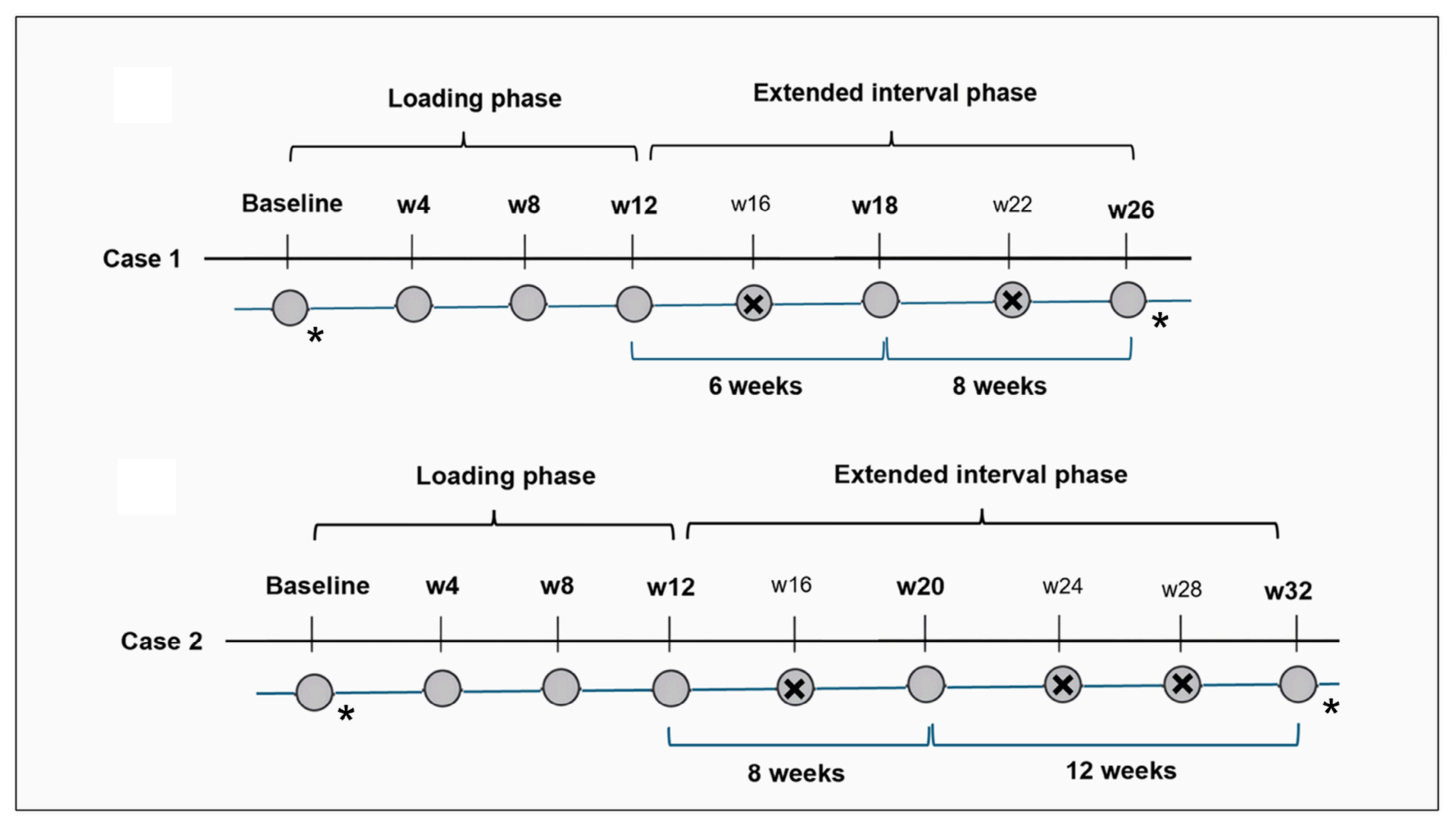
Treatment timelines and patient-reported outcome assessments in the two presented cases. Schematic overview of the treatment timelines for the two presented cases. Both patients received four monthly intravitreal faricimab injections during the loading phase, followed by a treat-and-extend regimen with progressively extended dosing intervals according to disease activity. Grey circles (○) indicate intravitreal faricimab injections, whereas grey circles containing a black × (⊗) indicate patient-reported outcome assessments, including the NEI VFQ-25 and the exploratory colour vividness item. Clinical and patient-reported outcomes were evaluated at baseline and after completion of six intravitreal faricimab injections (Week 26 in Case 1 and Week 32 in Case 2) with an asterisk (*) indicating the timing of NEI VFQ-25 administration.

The overall treatment schedule, timing of clinical examinations, intravitreal faricimab injections, and patient-reported outcome assessments for both cases are summarized in Figure 3.

## 4. Discussion

The most notable finding of the present report was the marked discordance between conventional clinical outcomes and patient-reported visual functioning. The patient with persistently poor visual acuity and residual structural abnormalities on OCT reported substantial improvements in both vision-related QoL and subjective colour experience at follow-up, whereas the patient with favourable anatomical and functional outcomes reported deterioration in both measures. Interestingly, these contrasting findings were accompanied by strikingly different descriptions provided by the patients of their visual experience, ranging from “like night and day” to a perception that “things had faded”. Because the present report compares eye-specific clinical outcomes with a patient-level PROM, complete agreement between these outcome measures should not necessarily be expected [10,17,18]. The NEI VFQ-25 assesses patients’ overall vision-related quality of life in everyday life rather than eye-specific visual function and may therefore capture aspects of visual experience beyond anatomical or functional changes in the treated eye alone [14,15]. Although based on only two patients, these observations suggest that subjective visual experience may differ substantially from that reflected by conventional clinical endpoints [11,12]. In particular, reductions in CRT and improvement in foveal contour do not necessarily indicate restoration of overall retinal integrity; persistent structural abnormalities of the outer retinal layers may continue to affect visual function despite otherwise favourable anatomical changes on OCT. This may be particularly relevant in older adults, for whom the consequences of visual impairment extend beyond retinal morphology and visual acuity to affect independence, social participation, emotional well-being, and overall QoL [1,3,8,9,13,19].

Discordance between clinical outcomes and patient-reported visual functioning has previously been reported in nAMD [11,12]. The NEI VFQ-25 has demonstrated responsiveness to changes in BCVA in nAMD populations, although the relationship between changes in these measures does not imply corresponding changes at the individual-patient level [20]. While the NEI VFQ–25 remains one of the most widely used PROMs in ophthalmology, perceived colour vividness is not assessed as a separate domain [14,15]. Although changes in colour perception have been reported in AMD, they have more commonly been evaluated using objective functional testing than through patient-reported assessment of perceived colour vividness [21]. Interestingly, the discordance observed in the present report was not limited to the relationship between clinical outcomes and patient-reported visual functioning.

Differences were also observed between the patterns of the exploratory colour-vividness ratings and NEI VFQ-25 item 12, which contributes to the Color Vision subscale. Importantly, item 12 does not directly assess the broader subjective colour experience addressed by the exploratory item, which encompasses perceived colour vividness, clarity, saturation, and the ability to distinguish between similar colours. Instead, item 12 assesses vision-related difficulty in selecting and matching clothing. The two items therefore differ substantially in their conceptual content and should not be regarded as alternative measures of the same construct. Their incomplete correspondence may reflect differences in item content, wording, response structure, or measurement variability. Accordingly, the observations in these two cases are descriptive and hypothesis-generating and do not establish that the exploratory item captures a distinct clinical domain not represented by the NEI VFQ-25.

Differences in baseline visual status may also contribute to the discordance between conventional clinical outcomes and patient-reported visual functioning, as patients with more severe impairment may perceive greater benefit from relatively small improvements or stabilization [8,19]. In Case 1, the small change in BCVA from 20/200 to 20/180 should be interpreted cautiously and cannot be considered to exceed the expected test–retest variability of Snellen visual-acuity measurement [22]. Partial spontaneous resorption of the submacular haemorrhage before treatment initiation should also be considered when interpreting the clinical course of Case 1. Importantly, however, the purpose of the present report was not to attribute changes in BCVA or retinal morphology exclusively to faricimab, but to examine the relationship and potential discordance between conventional clinical findings and patient-reported visual functioning during treatment.

The principal limitations of this report are inherent to its intentionally illustrative and hypothesis-generating design. The two contrasting cases were deliberately selected from the prospective cohort because they demonstrated marked discordance between conventional clinical and patient-reported outcomes; consequently, they are not intended to estimate the frequency or prevalence of this phenomenon or to permit comparative or causal inference. Eye-specific monocular clinical measures were compared with person-level NEI VFQ-25 outcomes, which may also be influenced by the fellow eye and by factors beyond retinal morphology and visual acuity [10,17,18].

The exploratory colour-vividness item was a non-validated ordinal measure combining several aspects of subjective colour experience, with numerical values from 0 to 100 representing ordered response categories with assessment-specific reference points rather than a continuous scale. No objective colour-vision, contrast-sensitivity, or reading-function testing was performed; therefore, the exploratory rating should be interpreted solely as a description of subjective colour experience rather than objective visual function.

Although the fellow eyes remained clinically stable and no clinically relevant progression of lenticular or other media opacity was identified, their potential influence on person-level subjective outcomes cannot be entirely excluded. Psychological, cognitive, systemic, and social factors were assessed through routine clinical history and patient reporting rather than dedicated standardized instruments, and expectation or interviewer effects cannot be excluded. The conventional NEI VFQ-25 scoring approach also has recognized psychometric limitations, including multidimensionality and the use of raw ordinal scores [23].

Individual changes in NEI VFQ-25 scores should also be interpreted cautiously because of potential measurement variability and floor or ceiling effects, and their clinical significance at the individual-patient level remains uncertain. The cases differed in treatment duration, partial spontaneous resorption of the submacular haemorrhage occurred before treatment initiation in Case 1, and detailed angiographic characterization and MNV subtype classification were unavailable. These limitations restrict interpretation beyond the two cases presented but do not detract from their intended role as illustrative, hypothesis-generating observations.

Nevertheless, these contrasting cases illustrate that favourable conventional clinical outcomes and favourable patient-reported visual functioning may not always occur in parallel and highlight the importance of considering the patient’s perspective alongside conventional clinical outcomes in nAMD.

## 5. Conclusions

These contrasting cases illustrate that changes in conventional clinical outcomes and patient-reported visual functioning may follow markedly different patterns in individual patients with nAMD. The exploratory colour-vividness item captured contrasting patterns in patients’ broader subjective colour experience that were consistent with the direction of change in overall patient-reported visual functioning in these two illustrative cases. These observations are not intended to establish causality or the validity or clinical utility of the exploratory item, but rather to highlight a potentially relevant aspect of subjective visual experience that warrants further investigation. Importantly, the patient’s perception of visual benefit represents a distinct perspective on treatment experience and cannot be inferred solely from anatomical or visual-acuity outcomes. As hypothesis-generating observations, these cases support further investigation of how subjective visual experience relates to established clinical and patient-reported outcomes during long-term nAMD treatment.

## Figures and Tables

**Table 1:** Summary of clinical and patient-reported characteristics of the two illustrative cases.

Characteristic	Case 1	Case 2
Age/sex	Seventies/F	Seventies/M
Treated eye	OD	OD
Fellow eye baseline BCVA (Snellen)	20/25	20/25
Fellow eye follow-up BCVA (Snellen)	20/25^−2^	20/25^−1^
Baseline BCVA	20/200	20/100
Follow-up BCVA	20/180	20/45
Baseline OCT/CRT, µm	480	341
Follow-up OCT/CRT, µm	389	186
Number of faricimab injections	6	6
Follow-up at sixth injection	Week 26	Week 32
Baseline NEI VFQ-25 composite score	33.87	67.08
Follow-up NEI VFQ-25 composite score	60.99	33.17
Baseline in Colour Vision item NEI VFQ-25	50	100
Follow-up in Colour Vision item NEI VFQ-25	75	50
Baseline colour-vividness rating	25	75
Follow-up colour-vividness rating	100	25
Patient-reported description	“Like night and day”	“Things had faded”

Abbreviations: BCVA, best-corrected visual acuity (Snellen); CRT, central retinal thickness; F, female; M, male; NEI VFQ-25, National Eye Institute Visual Function Questionnaire–25; OCT, optical coherence tomography; OD, right eye.

## Data Availability

The data presented in this study are available from the corresponding author upon reasonable request. The data are not publicly available due to privacy and ethical restrictions.

## Supplementary Materials

The following supporting information can be downloaded at: https://www.mdpi.com/article/10.3390/vision10040060/s1, Supplementary Table S1. Baseline and Follow-Up NEI VFQ-25 Subscale and Composite Scores in Cases 1 and 2. NEI VFQ-25 scores are presented on a 0–100 scale, with higher scores indicating better self-reported visual functioning. The composite score represents the arithmetic mean of the applicable vision-targeted subscale scores calculated according to the validated NEI VFQ-25 scoring procedure. *General Health and Driving are reported for completeness but are not included in the calculation of the NEI VFQ-25 composite score. General Health values are shown in parentheses to visually distinguish them from the subscale scores contributing to the composite score—indicates that the subscale score was not applicable and was therefore not calculated. **Abbreviation:** NEI VFQ-25, National Eye Institute Visual Function Questionnaire–25.

## Abbreviations

nAMD	neovascular age-related macular degeneration
QoL	quality of life
anti-VEGF	anti-vascular endothelial growth factor
OCT	optical coherence tomography
PROM	patient-reported outcomes
NEI VFQ-25	National Eye Institute Visual Function Questionnaire–25
BCVA	best-corrected visual acuity
CRT	central retinal thickness
