# Supplementary material for: Discordance Between Eye-Specific Clinical Outcomes and Patient-Reported Visual Function After Faricimab Treatment for Neovascular Age-Related Macular Degeneration: A Two-Case Report
Source: Vision (Basel). 2026 Aug 26;10(4):60. doi: 10.3390/vision10040060 (PMC13600179; doi:10.3390/vision10040060)
Supplement: Supplementary file 1 [file vision-10-00060-s001.zip › vision-4477255-supplementary.pdf]

Supplementary Table S1. Baseline and Follow-Up NEI VFQ-25 Subscale and Composite Scores in Cases 1 and 2

NEI VFQ-25 score	Case 1 Baseline	Case 1 Follow-up	Case 2 Baseline	Case 2 Follow-up
*General Health**	*(25)*	*(50)*	*(25)*	*(25)*
General Vision	20	60	50	40
Ocular Pain	50	75	87.50	62.50
Near Activities	33.33	58.33	58.3	25
Distance Activities	33.33	58.33	75	41.67
Social Functioning	50	75	87.50	37.50
Mental Health	6.25	50	50	12.50
Role Difficulties	12.50	75	62.50	12.50
Dependency	8.33	33.33	75	25
*Driving**	--	--	--	--
Color Vision	50	75	100	50
Peripheral Vision	75	50	25	25
**Composite score**	**33.87**	**60.99**	**67.08**	**33.17**

NEI VFQ-25 scores are presented on a 0–100 scale, with higher scores indicating better self-reported visual functioning. The composite score represents the arithmetic mean of the applicable vision-targeted subscale scores calculated according to the validated NEI VFQ-25 scoring procedure.

* General Health and Driving are reported for completeness but are not included in the calculation of the NEI VFQ-25 composite score. General Health values are shown in parentheses to visually distinguish them from the subscale scores contributing to the composite score. — indicates that the subscale score was not applicable and was therefore not calculated.

Abbreviation: NEI VFQ-25, National Eye Institute Visual Function Questionnaire-25.
